# Changes induced by parental neighboring touch in the clonal plant *Glechoma longituba* depend on the light environment

**DOI:** 10.3389/fpls.2024.1358924

**Published:** 2024-05-14

**Authors:** Linya Xing, Jiaxin Quan, Shuqi Zhang, Xiao Liu, Hua Bai, Ming Yue

**Affiliations:** ^1^ Key Laboratory of Resource Biology and Biotechnology in Western China, Ministry of Education, Northwest University, Xi’an, China; ^2^ Xi’an Botanical Garden of Shaanxi Province, Institute of Botany of Shaanxi Province, Xi’an, China

**Keywords:** clonal plant, parental neighboring touch, thigmomorphogenetic, shade response, maternal effect, light adaptability

## Abstract

**Introduction:**

Touch by neighboring plants is a common but overlooked environmental variable for plants, especially in dense vegetation. In addition, shade is inevitable for understory plants. The growth performance of clonal plant to the interaction between thigmomorphogenesis and shade response, and their impact on light adaptability is still unknown.

**Methods:**

At the present study, parental ramets of *Glechoma longituba* were exposed to two conditions (neighboring touch and shade), and their offspring ramets were in ambient or shaded environment. The phenotype and growth of parental and offspring ramets were analyzed.

**Results:**

The results showed that neighboring touch of parental ramets regulated the performance of offspring ramets, while the effect depended on the light environment. The parental neighboring touch occurring in ambient environment suppressed the expansion of leaf organ, showed as a shorter petiole and smaller leaf area. Moreover, *G. longituba* exhibited both shade avoidance and shade tolerance characters to shaded environment, such as increased leaf area ratio and leaf mass ratio, longer specific petiole length and specific stolon length. It was notable that these characters of shade response could be promoted by parental neighboring touch to some extent. Additionally, parental light environment plays an important role in offspring growth, parent with ambient light always had well-grown offspring whatever the light condition of offspring, but the growth of offspring whose parent in shaded environment was inhibited. Finally, for the offspring with shaded environment, the touch between parental ramets in shade environment showed a disadvantage on their growth, but the influence of the touch between parental ramets in ambient environment was slight.

**Discussion:**

Overall, the interaction of parental neighboring touch and shade environment complicate the growth of understory plants, the performance of plants is the integrated effect of both. These findings are conducive to an in-depth understanding of the environmental adaptation of plants.

## Introduction

Plants in dense vegetation compete for resources and optimize their growth based on neighbor detection cues. Accordingly, neighbor detection and response strategies are important mediators of interactions among species, which plays significant roles in plant coexistence and community assembly (de Wit et al., 2012; Pierik et al., 2013). The underlying mechanism of interactions among plants is related to light quality variation (Crepy and Casal, 2015; Zhang et al., 2020), root chemicals (Semchenko et al., 2014; Kong et al., 2018), airborne volatile organic compounds (Baldwin et al., 2006; Ninkovic et al., 2019), and mechanical stimuli caused by neighboring plants (Elhakeem et al., 2018; Douma and Anten, 2019). Among these, mechanical stimuli exist commonly in nature but are often ignored (Li and Gong, 2011). Except for stimuli from neighboring touch, mechanical stimuli are also related to wind, insect herbivory, heavy rains, buzzing bees, tree strangling, acoustic vibration, and the navigation of roots around obstacles (Monshausen and Haswell, 2013; Brenya et al., 2022). As one of the environmental factors, mechanical stimuli have been shown to induce a range of anatomical, physiological, morphological, and developmental responses, termed thigmomorphogenesis (Jaffe, 1973). Thigmomorphogenetic plants typically have a reduced leaf area, petiole length, height, and stem length, and enhanced flexural rigidity in stem and roots; these make plants more resistant to mechanical stimuli and more likely to survive in stressful environments (Liu et al., 2007; Braam and Chehab, 2017). Significantly, the touch signal of neighboring leaf tips occurs before light signals and appears to be the earliest means of aboveground plant–plant signaling (de Wit et al., 2012). As a unique way for plants to rapidly detect future competitors, the touch by neighbors may play an important role in the environmental adaptability of plants.

Understory plants employ two different strategies to cope with shade conditions: shade avoidance syndrome (SAS) and shade tolerance syndrome (STS). Although both will optimize light capture and utilization by increased specific leaf area (SLA) and photosystem II and I ratio (PSII : PSI), and reduced chlorophyll a:b ratio, the plants with STS appear to have a slight elongation in low light (Valladares and Niinemets, 2008). Plants with SAS mainly manifested a suite of traits to grow towards the light including stem and petiole elongation reaction and leaves bending upward (Fraser et al., 2016; Fiorucci and Fankhauser, 2017; Xu et al., 2021; Martinez-Garcia and Rodriguez-Concepcion, 2023). The overlap between two strategies is due to the common signaling components associated with photoreceptors and phytohormone (Gommers et al., 2013). For example, phytochrome-mediated signaling pathways trigger the increase of gibberellins in shading plants, subsequently promoting stem and petiole elongation (Pierik et al., 2004; Ballare and Pierik, 2017). In contrast, the reduction of gibberellins is a key factor of phenotype change induced by thigmomorphogenesis (Boernke and Rocksch, 2018; Telewski, 2021; Wang et al., 2023). Therefore, the change in gibberellins level and the corresponding growth response of thigmomorphogenesis and SAS usually occur in opposite directions (Anten et al., 2005). However, for dense understory vegetation, the touch by neighbors and the shaded environment often coexist. Accordingly, plant performance is the result of the interaction of both. There have been several studies involving the interaction between mechanical stimuli and shade, but the results were different. Some studies showed that competition for light in dense vegetation suppressed thigmomorphogenesis because any reduction in height growth associated with thigmomorphogenesis resulted in reduced fitness (Sterck and Bongers, 1998; Henry and Thomas, 2002), while others did not find any thigmomorphogenesis inhibition for plants in dense stands (Liu et al., 2007). As such, the interaction of these two conditions is still unknown, and more research is required to thoroughly understand the growth of understory plants.

In addition, the performance of plants is also affected by the environment of their parents (Galloway and Etterson, 2007; Marshall and Uller, 2007; Dong et al., 2019), which regulates the phenotype, growth, and life history strategies of the offspring so that they may pre-adapt to the future environment (Galloway, 2005; Wang et al., 2022). This effect of parental environment on their offspring was termed “maternal effect”. This impact is not transmitted through genetic inheritance but rather through the environment or other non-genetic factors provided by the parent during the developmental and reproductive processes (Galloway, 2005; Marshall and Uller, 2007). Many studies indicate that maternal effects could persist in the offspring. For instance, compared to the *Polygonum persicaria* offspring of sun-grown parents, the offspring of parents with a shaded environment produced greater leaves, larger SLA, higher total leaf area and biomass, and greater fitness (Galloway and Etterson, 2007; Baker et al., 2018). In addition, maternal effects induced light adaptability of *Campanula americana* offspring by directly influencing their germination rate and season (Galloway, 2005). In summary, the growth of offspring is often affected by the light environment of the parents. To our knowledge, there are very few reports on the maternal effects of the interaction between neighboring touch and shade.

In the present study, the interaction of parental neighboring touch and shade was explored with the clonal herb *Glechoma longituba* (Nakai) Kuprian, a “faithful” transmitter of the maternal effect (Guo et al., 2022; Tie et al., 2022). Two environmental factors (light and touch) were conducted on the parental ramets. By analyzing the growth parameters of parental ramets and their offspring, the following questions were addressed: (a) Does the effect of parental neighboring touch vary with a light environment? (b) If SAS occurs, what is the result of the interaction between thigmomorphogenesis and SAS? (c) Is the effect of parental light environment on clonal offspring affected by parental neighboring touch? We predicted that the impact of parental neighboring touch on clonal plants was linked to their light environment, which also influenced the maternal effect on environmental adaptability of clonal offspring.

## Materials and methods

### Plant material and propagation

**Figure 1:**
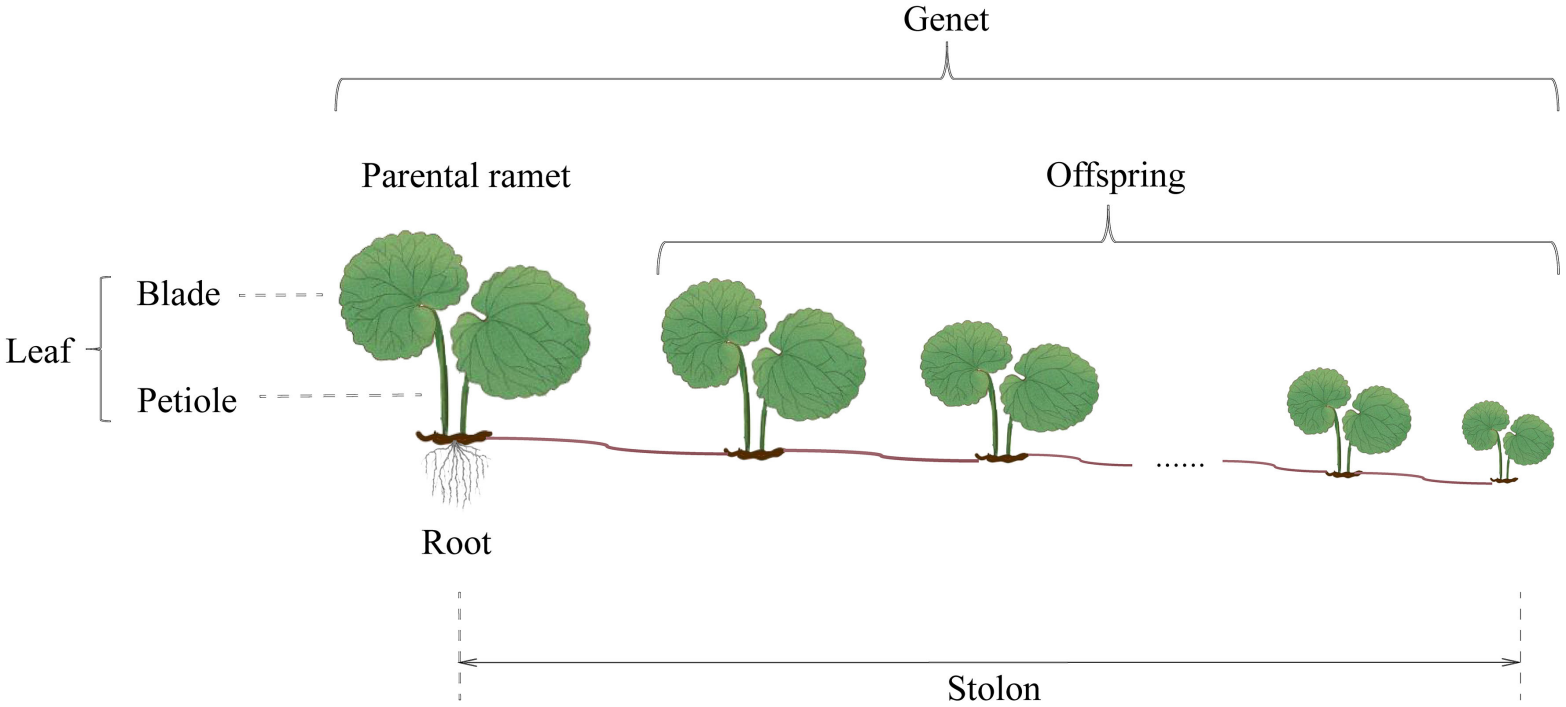
Clonal growth pattern of *Glechoma longituba* (Nakai) Kuprian.

*Glechoma longituba* (Nakai) Kuprian is a perennial herb widely distributed in the forest, on the roadside, and by creeks of tropical, subtropical, and temperate areas in China. The monopodial stolons are able to creep on the ground, and ramets can develop on each stolon node (Quan et al., 2021). Each ramet produces two petioles and leaves. The blades are heart-shaped, with margins bearing rounded teeth (**Figure 1**). *G. longituba* has high phenotypic plasticity in response to light (Liu et al., 2015; Tie et al., 2022). In addition, for the rapid clonal propagation, *G. longituba* forms a complex ramet network; touch between ramets was inevitable.

The *G. longituba* in our experiment was collected from Jiwozi in the Qinling Mountains, Shaanxi, China. To ensure the uniformity of the genotypes and reduce the impact of the previous environment, the plant materials were collected from the same genet and then were vegetatively propagated for at least 4 months in a greenhouse at Northwest University in Xi’an (397 m a.s.l., 34.3°N, 108.9°E).

A total of 96 healthy and similarly sized (0.78 g ± 0.29 g) ramets were selected as parental ramets, and then were divided into 48 ramet pairs randomly. Each ramet pair was transplanted into a plastic pot (55 cm length × 28 cm width × 5.5 cm depth). The peat soil, perlite, and vermiculite (4:1:1, v/v/v) were mixed and utilized as the culture soil. The greenhouse conditions were a 24/20°C day/night temperature cycle, a 12/12-h light/dark cycle, and a 300 μmol m^−2^ s^−1^ photosynthetic photo flux density (PPFD) during the light cycle. PPFD levels were measured with a Quantum Metre (LQS-QM, Apogee Instruments Inc., USA). The relative humidity was maintained at 40%. Plants were watered into the soil directly every 3 days throughout the experiment and researchers avoided the droplets touching the leaves during watering. To minimize the effects of microenvironment variation in the greenhouse, the position of each pot was changed weekly.

### Experimental design

**Figure 2:**
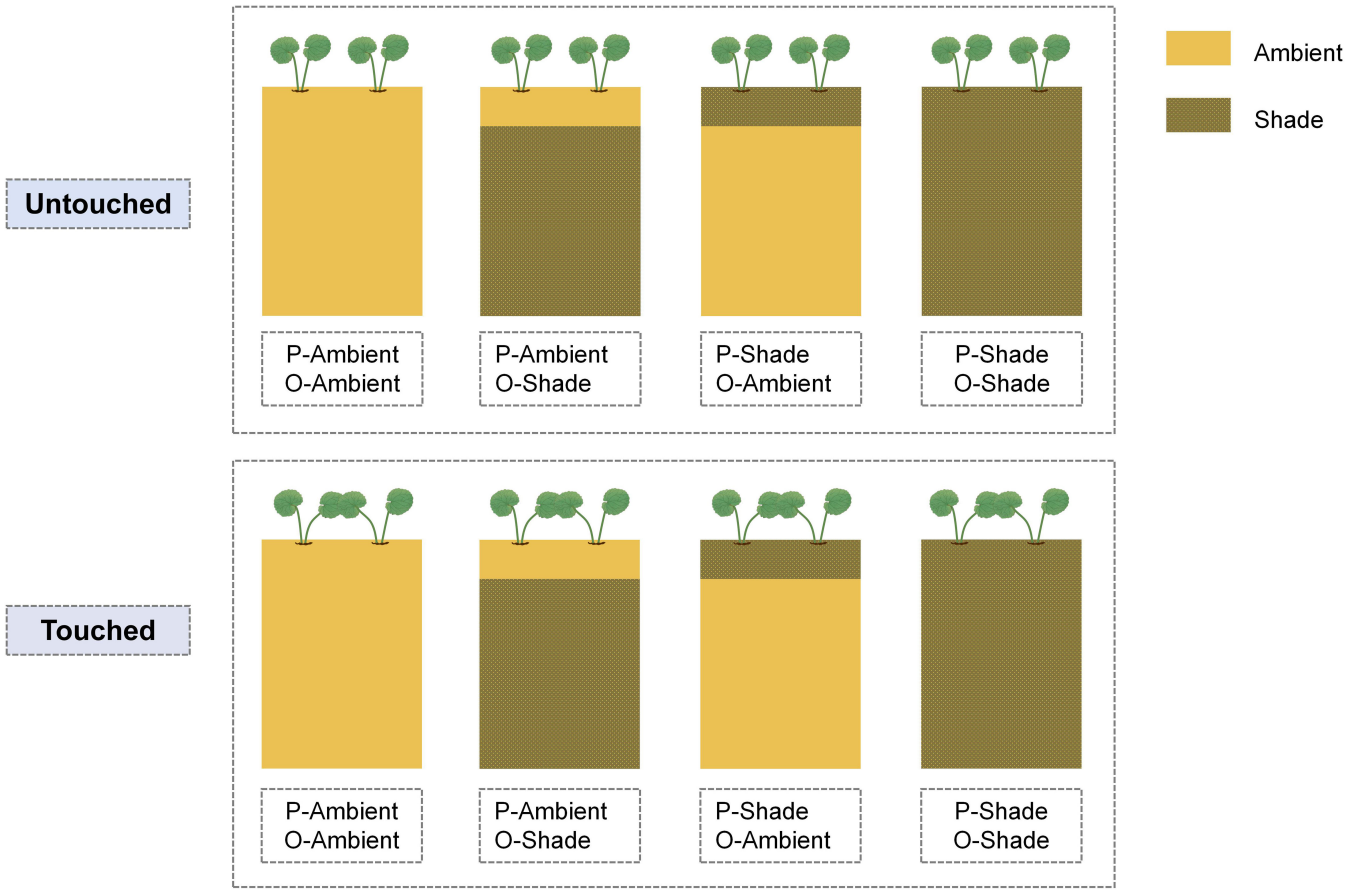
Schematic of the experiment. P-ambient: parents grew in an ambient environment; P-shade: parents grew in a shaded environment; O-ambient: offspring grew in an ambient environment; O-shade: offspring grew in a shaded environment. Touched: neighboring parental ramets were waved with a thin wooden stick gently to make two blade touch; Untouched: no touch happened between a pair of parental ramets.

After a week of transplanting, a 45-day experiment was conducted from 1 November 2022 to 15 December 2022. At first, 48 parental ramet pairs were divided into two groups randomly; half was placed under ambient light conditions, and the rest was placed in a shaded environment. Then, half of each group was subjected to touch treatment, and the other had no action. During the experiment, the newborn offspring was placed in an ambient light environment or under shade conditions (**Figure 2**). Accordingly, the experiment included a total of eight treatments (two parental light conditions × two parental neighboring touch treatments × two offspring light conditions). Each treatment was designed with six replicates.

### Shading treatment

The shaded environment was achieved by hanging a shading net 20 cm above the top of ramets. The PPFD was about 80–100 μmol m^−2^ s^−1^ in the shaded environment. During the experiment, according to the experiment’s design, if the parent and offspring were in different light environments, the shading treatment was only performed above the parent or offspring.

### Parental neighboring touch treatment

The touch treatment was conducted between parental ramet pairs. Two petioles of the neighbor parental ramets were waved with a thin wooden stick gently to make the two leaf blades touch. During the treatment, we tried to minimize the bending angle of the petiole to avoid damage. The touch treatment was only performed on parental ramets and continued for 45 days. The touch was handled twice a day at 9:00 a.m. and 6:00 p.m., respectively; three contacts were made each time. To ensure the uniform touch intensity during repeated action, all the touch treatments were controlled by the same person. Furthermore, during the experiment, the distance between parental ramet pairs was maintained at 5 cm, a distance that is long enough for leaves of ramet pairs to not touch each other naturally during growth except for human-facilitated touch.

### Measurement of growth parameters

At the end of the experiment, we performed growth architecture and biomass measurements. For parents, the blade area and petiole length were first measured. Then, biomass indexes were measured, including total biomass, aboveground biomass (leaf biomass and node biomass), and root biomass. Finally, the following indicators of parental ramets were estimated: SLA (leaf area per leaf mass), specific petiole length (SPL; length of petiole per petiole biomass), leaf area ratio (LAR; blade area per total biomass), and leaf mass ratio (LMR; blade biomass per total biomass).

For offspring, the length of the longest stolon, total blade area, and the number of total ramets were recorded. After that, total biomass, leaf biomass, and stolon biomass were measured. The following indicators were estimated: specific stolon length (SSL; length of stolon per stolon biomass), stolon biomass/leaf biomass (S/L), LAR (blade area per total biomass), and LMR (blade biomass per total biomass). The comparison table of abbreviations is shown in **Table 1**.

**Table 1:** Abbreviation comparison.

Abbreviation	Complete spellings
SAS	Shade avoidance syndrome
STS	Shade tolerance syndrome
SLA	Specific leaf area
SPL	Specific petiole length
SSL	Specific stolon length
R/S	Root biomass/shoot biomass
S/L	Stolon biomass/leaf biomass
LAR	Leaf area ratio
LMR	Leaf mass ratio

Length of petiole and stolon was measured with a vernier caliper and tapeline, respectively. Blade area was calculated with Motic software (Motic Images Plus 2.0. Ink, Motic, China) after being scanned with a scanner (Perfection V19, EPSON, China). Biomass was measured after the samples were dried at 80°C for 48 h to a constant weight with an electronic balance (Sartorius BT25S, Beijing, China).

### Statistical analysis

Before statistical analyses, to meet the assumptions of homoscedasticity and normality, the following data were subjected to logarithmic transformation: total biomass and root biomass of parent, and total biomass, stolon biomass, and S/L of offspring. We analyzed the integrated differences of phenotypic and growth among treatments of parental ramets and offspring, respectively, using a PERMANOVA (McArdle and Anderson, 2001), where parental neighboring touch treatment (NT; touch or not), parental light environment (PL; ambient or shade), offspring conditions (ambient or shade) and their interactions, and the covariates (initial fresh weight of parental ramet) were used as predictors, estimating the significance value by 999 permutations. Then, we conducted ANCOVAs to analyze the effects of PL, offspring light environment (OL), NT, and interaction regimes on all traits with parental fresh weight as a covariate. The LSD test was chosen as the method of multiple comparisons to test the significance among different treatments. Any effect of parental conditions, either direct (PL or NT) or in interaction with the offspring conditions (PL × OL, NT × OL, PL × NT × OL) that remained significant after removing the linear part of the maternal investment (initial fresh weight of parent), was considered a maternal effect (Bolker et al., 2009; Galloway et al., 2009; Puy et al., 2022). All data analyses and diagrams were carried out using R (v.3.2.3, R Core Team, 2016) with α = 0.05 as significance level. The images were further processed with Adobe Illustrator (v 28.1, Adobe Systems Incorporated, 2023).

## Results

The PL, OL, NT, the interaction of PL and OL (PL × OL), and the interaction of PL and NT (PL × NT) significantly affected the growth of parental ramets and offspring. Nevertheless, the interaction of OL and NT (OL × NT) and three factors (PL × OL × NT) had no effect on them. Among three factors, PL displayed the most dissimilar traits of *G. longituba* among treatments (*R* ^2^ = 0.297 for parent and *R* ^2^ = 0.519 for offspring). For the parent, the next important factor was OL (*R* ^2^ = 0.167), followed by PL × NT (*R* ^2^ = 0.075), PL × OL (*R* ^2^ = 0.071), and NT (*R* ^2^ = 0.068). For the offspring, OL (*R* ^2^ = 0.173), NT (*R* ^2^ = 0.048), PL × OL (*R* ^2^ = 0.036), and PL × NT (*R* ^2^ = 0.032) played significant effects sequentially (**Supplementary Table S1**).

### The influence of parental neighboring touch on parental ramets in different light environments

For parental ramets, there were significant effects of PL, OL, and NT on biomass (including total, aboveground, and root biomass). Additionally, PL affected SLA, SPL, LAR, and R/S; OL showed an effect on the blade area, SPL, LAR, LMR, and R/S, while NT influenced SLA, LAR, and petiole length; the interaction of PL and OL affected the blade area, petiole length, LMR, LAR, and R/S significantly; PL × NT influenced the blade area, SLA, and LAR. Moreover, OL × NT and PL × OL × NT did not show an effect on parents (**Table 2**).

**Table 2:** Three-way ANCOVAs for effects of parental light environment (PL), offspring light environment (OL), parental neighboring touch (NT), and their interactions on growth indicators of parental ramets.

Source of variation	df	Total biomass (g) ^a^		Aboveground biomass (g)		Root biomass (g) ^a^		R/S		Blade area (cm^2^)		SLA (cm^2^/g)		Petiole length (cm)		SPL (cm/g)		LAR(cm^2^/g) ^a^		LMR (g/g)	
		*F*	*p*	*F*	*p*	*F*	*p*	*F*	*p*	*F*	*p*	*F*	*p*	*F*	*p*	*F*	*p*	*F*	*p*	*F*	*p*
Fresh weight	1	6.993	**0.014**	4.508	**0.045**	7.484	**0.012**	2.195	0.152	0.541	0.470	3.642	0.069	0.028	0.869	4.701	**0.041**	5.830	**0.024**	0.637	0.433
Parental light environment (PL)	1	48.609	**<0.001**	32.886	**<0.001**	46.368	**<0.001**	23.781	**<0.001**	0.246	0.625	58.640	**<0.001**	0.303	0.588	9.997	**0.004**	77.721	**<0.001**	1.888	0.183
Offspring light environment (OL)	1	24.918	**<0.001**	11.064	**0.003**	51.234	**<0.001**	44.822	**<0.001**	7.510	**0.012**	0.693	0.414	<0.001	0.995	7.422	**0.012**	9.724	**0.005**	21.517	**<0.001**
Neighbor touch (NT)	1	7.121	**0.014**	5.333	**0.03**	6.016	**0.022**	0.291	0.595	0.147	0.704	17.118	**<0.001**	16.543	**<0.001**	0.317	0.579	12.841	**0.002**	0.401	0.533
PL×OL	1	0.406	0.530	0.806	0.378	1.863	0.186	15.859	**0.001**	9.461	**0.005**	2.257	0.147	9.250	**0.006**	0.147	0.704	6.867	**0.015**	10.420	**0.004**
PL×NT	1	0.329	0.572	0.029	0.866	0.077	0.784	0.228	0.638	22.088	**<0.001**	30.731	**<0.001**	4.150	0.053	1.425	0.245	35.503	**<0.001**	0.133	0.719
OL×NT	1	1.226	0.280	1.537	0.228	0.606	0.444	<0.001	0.997	0.696	0.413	2.411	0.134	0.232	0.634	1.542	0.227	2.519	0.126	0.001	0.999
PL×OL×NT	1	0.007	0.934	0.035	0.853	0.639	0.432	2.183	0.153	1.197	0.285	0.262	0.614	2.162	0.155	<0.001	0.997	0.613	0.442	0.975	0.334

Values in bold indicate significant effects (*p* < 0.05) of factors and their interactions. The lowercase letter “a” indicates that the data are log-transformed.The shading indicates that the p-value is significant.

**Figure 3:**
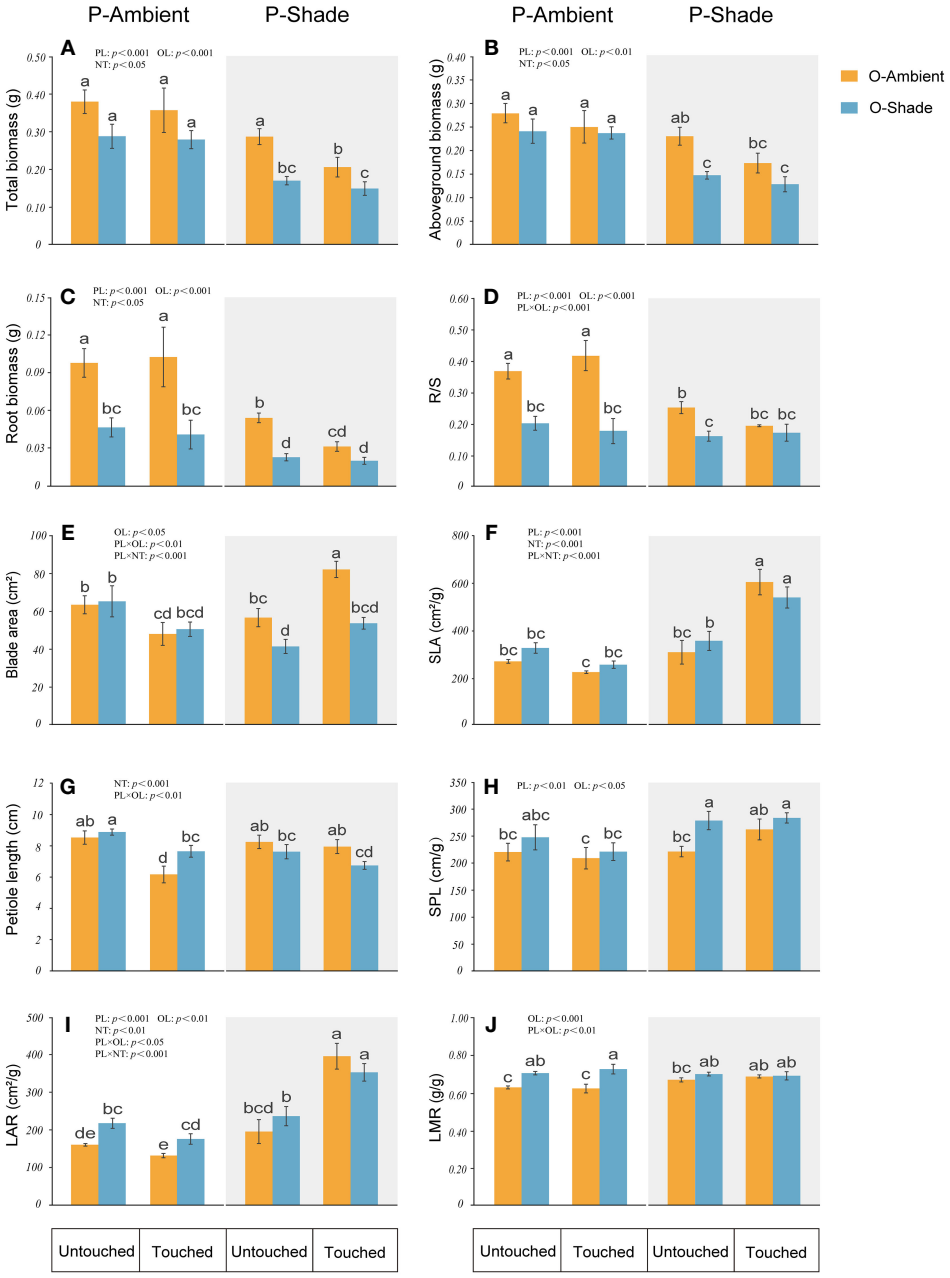
Growth of parental ramets in different treatments. **(A)** Total biomass; **(B)** aboveground biomass; **(C)** root biomass; **(D)** root biomass/shoot biomass (R/S); **(E)** blade area; **(F)** specific leaf area (SLA); **(G)** petiole length; **(H)** specific petiole length (SPL); **(I)** leaf area ratio (LAR); **(J)** leaf mass ratio (LMR). P-ambient: parents grew in an ambient environment; P-shade: parents grew in a shaded environment; O-ambient: offspring grew in an ambient environment; O-shade: offspring grew in a shaded environment. Different letters indicate significant difference among the treatments, and the same letter indicates no significant difference at the 0.05 level with the LSD test. Multiple comparisons of total biomass, root biomass, and LAR are based on log-converted data. Error bars show the SE.

When the genet (including parent and their offspring ramets) was in ambient light, neighboring touch (NT) mainly reduced the blade area and the petiole length of parental ramets but had no significant effect on other parental parameters. Additionally, compared with the ambient environment, the growth of genet in a shaded environment was depressed. Parental ramets displayed reduction in the blade area, biomass (aboveground, roots, and total biomass), and R/S, but increased SPL, LAR, and LMR. However, in the shaded environment, if touch between neighboring parental ramets happened, some traits induced by shade were changed. Upon recovery from the reduction in the parental blade area, SLA and LAR even became larger (**Figure 3**).

### The influence of parental neighboring touch on offspring ramets in different light environments

For the offspring ramets, PL and OL displayed effects on almost all traits involved in our study, except for OL on stolon biomass. Neighboring touch treatment (NT) had effects on most traits except for stolon biomass/leaf biomass and LMR. Moreover, the PL × OL interaction affected the blade area, leaf biomass, and SSL of the longest stolon; PL × NT influenced total biomass, stolon biomass, blade area, and stolon/leaf biomass, but OL × NT only affected leaf biomass. Same as the parent, PL × OL × NT did not show an effect on the offspring (**Table 3**).

**Table 3:** Three-way ANCOVAs for effects of parental light environment (PL), offspring light environment (OL), parental neighboring touch (NT), and their interactions on growth indicators of offspring ramets.

Source of variation	df	Total biomass (g) ^a^		Stolon biomass (g) ^a^		Leaf biomass (g)		Stolon biomass/Leaf biomass ^a^		Total blade area (cm^2^)		Number of ramets		Length of the longest stolon (cm)		SSL of the longest stolon (cm/g)		LAR (cm^2^/g)		LMR (g/g)	
		*F*	*p*	*F*	*p*	*F*	*p*	*F*	*p*	*F*	*p*	*F*	*p*	*F*	*p*	*F*	*p*	*F*	*p*	*F*	*p*
Fresh weight	1	5.603	**0.027**	1.562	0.224	8.296	**0.008**	2.411	0.134	1.889	0.183	14.027	**0.001**	4.336	**0.049**	8.884	**0.007**	0.036	0.851	2.536	0.125
Parental light environment (PL)	1	210.375	**<0.001**	221.496	**<0.001**	92.739	**<0.001**	8.832	**0.007**	65.724	**<0.001**	82.482	**<0.001**	107.583	**<0.001**	338.294	**<0.001**	17.211	**<0.001**	10.370	**0.004**
Offspring light environment (OL)	1	31.672	**<0.001**	2.704	0.114	37.770	**<0.001**	27.823	**<0.001**	58.122	**<0.001**	23.117	**<0.001**	10.137	**0.004**	255.398	**<0.001**	44.476	**<0.001**	30.298	**<0.001**
Neighbor touch (NT)	1	21.647	**<0.001**	10.552	**0.004**	13.145	**0.001**	2.184	0.153	31.515	**<0.001**	9.292	**0.006**	21.820	**<0.001**	23.768	**<0.001**	12.487	**0.002**	2.155	0.156
PL×OL	1	4.134	0.054	3.903	0.06	8.334	**0.008**	0.131	0.720	9.886	**0.005**	17.531	**<0.001**	0.675	0.420	62.936	**<0.001**	0.102	0.752	0.116	0.737
PL×NT	1	6.757	**0.016**	15.056	**0.001**	1.412	0.247	4.472	**0.045**	5.444	**0.029**	2.388	0.136	3.863	0.062	0.486	0.493	4.056	0.056	4.193	0.052
OL×NT	1	0.596	0.448	0.622	0.439	9.095	**0.006**	3.517	0.073	3.557	0.072	5.795	**0.024**	1.783	0.195	1.756	0.198	0.002	0.969	3.410	0.078
PL×OL×NT	1	0.027	0.872	0.580	0.454	2.010	0.170	1.837	0.188	0.28	0.602	6.180	**0.021**	0.067	0.798	0.178	0.677	0.651	0.428	1.889	0.183

Values in bold indicate significant effects (*p* < 0.05) of factors and their interactions. The lowercase letter “a” indicates that the data are log-transformed.The shading indicates that the p-value is significant.

**Figure 4:**
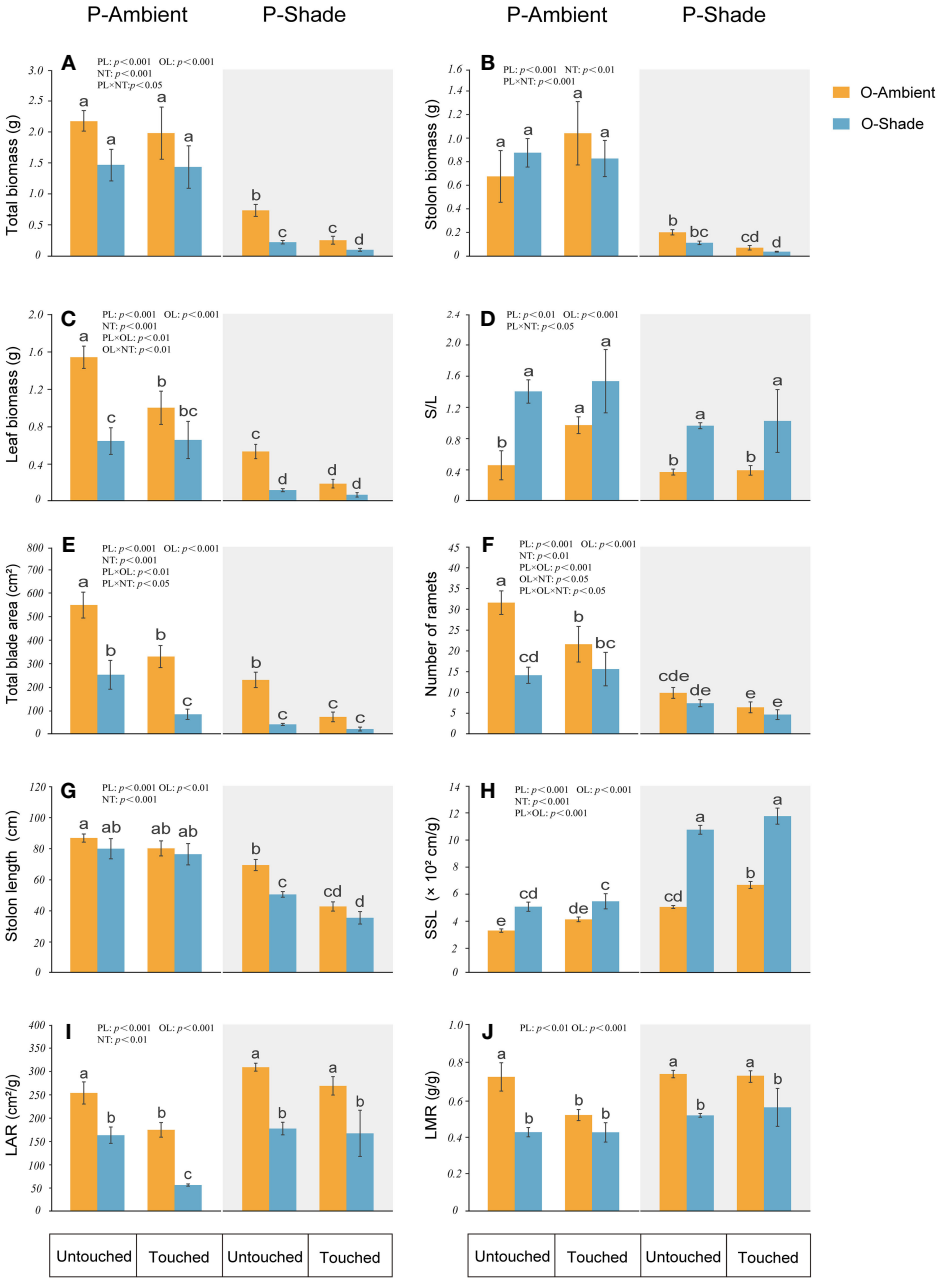
Growth of offspring ramets in different treatments. **(A)** Total biomass; **(B)** stolon biomass; **(C)** leaf biomass; **(D)** stolon biomass/leaf biomass (S/L); **(E)** total blade area; **(F)** number of ramets; **(G)** length of the longest stolon; **(H)** specific stolon length (SSL); **(I)** leaf area ratio (LAR); **(J)** leaf mass ratio (LMR). P-ambient: parents grew in an ambient environment; P-shade: parents grew in a shaded environment; O-ambient: offspring grew in an ambient environment; O-shade: offspring grew in a shaded environment. Different letters indicate significant differences among the treatments, and the same letter indicates no significant differences at the 0.05 level with the LSD test. Multiple comparisons of total biomass, stolon biomass, and stolon biomass/leaf biomass are based on log-converted data. Error bars show the SE.

When the genet was in ambient light, in offspring, parental neighboring touch decreased total leaf biomass and blade area, but enhanced S/L. When the genet was in a shaded environment, as compared with the ambient environment, shade leads to a reduction in biomass (stolon, leaf, and total biomass), the total blade area, and the length of the longest stolon. In contrast, there is an increase in SSL and S/L. However, if touch between neighboring parental ramets happened, it caused a greater decrease in the total biomass, stolon biomass, and length of the longest stolon than in those without touch (**Figure 4**).

### The influence of parental light environment on offspring light adaptability

We compared the offspring performance in the same light environment as their parents or in a different light environment (**Figure 4**). When the parental ramets were in ambient light, the offspring with a shaded environment did not show a significant difference from offspring with ambient light in the total biomass, stolon biomass, and length of longest stolon. However, they had a lower number of ramets, leaf biomass, blade area, LAR, and LMR, and higher SSL and stolon biomass/leaf biomass. Additionally, if the parental ramets are in a shaded environment, even if their offspring grew in an ambient environment, these offspring will still have a lower leaf, stolon, and total biomass, a smaller blade area, a shorter stolon length, but a larger SSL. In contrast to the offspring with ambient light, the offspring with a shaded environment will have the lowest total biomass, leaf biomass, blade area, LAR, LMR and stolon length, but the largest SSL.

### The effect of parental neighboring touch on offspring light adaptability

When parents grew in ambient light, the following traits of offspring with ambient light were changed by parental neighboring touch: leaf biomass, leaf area, ramet number, LAR, and LMR were decreased, and stolon biomass/leaf biomass was increased. Total biomass, stolon biomass, SSL, and stolon length did not change significantly. For the offspring with a shaded environment, touch just reduced leaf area and LAR; others were unchanged (**Figure 4**).

When parents grew in a shaded environment and offspring grew in ambient light, except for ramet number, stolon biomass/leaf biomass, LAR, and LMR without any changes, SSL was increased and all other traits decreased due to parental neighboring touch. For offspring with a shaded environment, however, leaf biomass, leaf area, ramet number, stolon biomass/leaf biomass, SSL, LAR, and LMR were not influenced by parental neighboring touch, with only total biomass and the length and biomass of stolon decreased (**Figure 4**).

## Discussion

Our findings reveal complex interactions between parental neighboring touch and light environments on the clonal plant *G. longituba*. Namely, the effect of parental neighboring touch changed with light conditions. When plants grew in ambient light, the reduction in leaf investment induced by touch could help plants minimize damage caused by mechanical stimuli. When plants grew in a shaded environment, SAS or STS response of plants was promoted by parental neighboring touch to some extent, which was conducive to survival in the shade. Moreover, the maternal effect of PL on light adaptability of offspring ramets also relied on parental neighboring touch. If touch occurred between parental ramets in ambient light, the shaded offspring was slightly affected, while the effect was disadvantageous when touch happened on parental ramets in the shaded environment.

### The effect of parental neighboring touch associated with light environment

In the present study, the growth of the clonal plant *G. longituba* was affected by the touch between neighbor parents and not only parental ramets but also their offspring ramets. However, the effect varied with a light environment. For example, in an ambient environment, it seems that the effect of parental neighboring touch was mainly on leaves; the reduction in the blade area and petiole length of the parental ramets was the most intense phenotypic change observed after direct touch by a neighbor, which is also regarded as the core morphological change of thigmomorphogenesis (Brenya et al., 2022). The compression of leaf and petiole was associated with the increases in cell wall stiffness and decreases in cell elongation, which is correlated with ethylene-regulated pectin degradation induced by touch (Wu et al., 2020). Being a clonal herb with a long stolon, the petiole is the vertical support of *G. longituba*; a shorter petiole limits the bending moment, and together with a smaller blade, they reduce the risk of various mechanical strains, such as plastic deformation, uprooting, and buckling, and are likely adaptive in crowded vegetation (Langer et al., 2021; Telewski, 2021; Langer et al., 2022).

Parental environmental signals that can be perceived by clonal offspring via a connected spacer (stolon or rhizome) have been proven (Liu et al., 2015; Guo et al., 2022; Tie et al., 2022; Xue et al., 2022). Moreover, in this study, although touch only took place between parents, its impact was transferred to their offspring, causing the decrease of ramet number, leaf area, leaf biomass and leaf investment (decreased LAR and LMR), but relatively less reduction in stolon biomass (increased S/L). The performance of offspring ramets induced by parental neighboring touch owing to clonal integration was also proved with *Leymus secalinus* (Sui et al., 2011). The sharing of environmental signals among interconnected ramets was considered to favor young ramets (Wei et al., 2019). In this study, to some extent, the compression of phenotype and growth, and more allocation to the stolon, those induced by touch benefits plants in withstanding mechanical stress, which is also the form of plants in high mountain and grazed areas to protect plants from the stresses caused by high wind, rain, physical touch from other plants, or trampling (Braam, 2005; Sui et al., 2011).

When *G. longituba* was in a shaded environment, growth was obviously inhibited, as shown by the lower biomass accumulation and ramet production, which was due to the limitation of light resources. Shade induced a significant impact on both leaf and stolon. Despite the decreased biomass, some changes in parental ramets caused by shade were in favor of adapting to low-light conditions. For instance, notwithstanding the smaller leaf area, more investment to leaf in parental ramets showed increased LAR and LMR, which optimized light capture in the shade. Moreover, the longer SPL in parents and SSL in offspring represented expansion in the vertical and horizontal directions, which suggested an effort of clonal plants to escape from the shade (**Figures 3**, **4**). Evidently, the clonal plant *G. longituba* exhibited both STA and SAS characters to tolerate the shaded environment.

Interestingly, if parental neighboring touch happened in the shade, it seems that some responses of *G. longituba* were more beneficial to survival in the shaded environments. For example, the reduction of parental leaf area in the shade was offset, and even more resources were allocated to the leaf area, causing larger SLA and more LAR (**Figure 3**). Additionally, although the length and biomass of stolon decreased, the long SSL and SPL was maintained the same as in the shade (**Figure 4**). In addition, the touch of the parent caused larger SLA in the shaded offspring, and these responses were not even represented in the shaded environment. Consequently, SAS or STS responses induced by shade were not restrained by parental neighboring touch and, instead, were promoted to some extent.

### Parental neighboring touch and maternal effect on offspring light adaptability

The connected stolon provides physical support for communication among ramet nets and guarantees the parental and ongoing effects on offspring ramets. The maternal effect and the light adaptation of clonal offspring induced by it have been discussed in many studies. Some documented that the phenotype induced by the maternal effect may facilitate offspring to pre-adapt to a light environment (Guo et al., 2022; Tie et al., 2022; Xue et al., 2022). The alternative view, however, stated that parental shading effects contributed little to the tolerance of clonal offspring to shading (Dong et al., 2019). Our results displayed that a parental high-light environment was in favor of offspring performance. If the parental ramets were in ambient light, their offspring always maintained a higher total biomass and ramet number regardless of OL. The assurance in total biomass of the shaded offspring was mainly related to stolons. By contrast, the offspring with shaded parents usually had a lower biomass even if they were under ambient light conditions. In brief, it seemed from our results that the offspring with a shaded environment did not benefit from the shading experience of their parent despite the matching parent–offspring environment. However, plants will avoid excessive growth or defense through a negative feedback–regulatory loop and achieve balance in response to adverse environments (Li et al., 2019), More precisely, the decrease in growth of *G. longituba* does not necessarily mean that they are completely unadaptable; resources may be more devoted to other traits not involved in the present study, such as defense and life span.

If parental ramets are in an ambient environment, the offspring with a shaded environment showed similar growth whether touch occured or not, and they both exhibited the same biomass (leaf, stolon, and total biomass), ramet number, LMR, SSL, and stolon biomass/leaf biomass. Instead, if touch happened when parents grew in a shaded environment, the biomass of offspring with a shaded environment was decreased, accompanied by a reduction in length and biomass of stolons. Of course, the longest SSL and a high S/L were still kept (**Figure 4**). These traits responded in opposite directions, suggesting the potential for complex trade-offs among traits. Therefore, the effects of parental neighboring touch on offspring under shaded conditions depended on PL. If light resource in the parental environment was abundant, the impact of parental neighboring touch on the offspring with a shaded environment was not obvious, whereas when the parent was under limited light conditions, touch from a neighbor was more disadvantageous to the growth of the clonal plant *G. longituba*. The interaction of mechanical stimuli and shade has been reported by several studies; some documented that shading decreased or eliminated the thigmomorphogenesis (Henry and Thomas, 2002), while others showed opposite results (Feng et al., 2019). These results disclosed a complexity of interaction between shade and mechanical stimuli on plant morphology, growth, and allocation. Note that the mechanical stimuli in these studies were simulated with wind; the effect not only is a single mechanical stimulus, but also affects the exchange of heat, water vapor, and CO_2_ around leaves (Feng et al., 2019), which made the findings more diverse. Our study focused on the effect of contact between neighboring leaves, which broadens our understanding of mechanical stimuli.

## Conclusion

Our results illustrated that the impact of parental neighboring touch on the clonal plant *G. longituba* was dependent on the light environment. For instance, in an environment with sufficient light, depressed leaf investment induced by touch was regarded as a trade-off to resist potential mechanical damage. In a shaded environment, SAS or STS response induced by shade was promoted by parental neighboring touch to some extent, which was conducive to the survival of plants in a shaded environment. If touch occurred on the parental ramets in ambient light, the light adaptability of the shaded offspring was slightly affected, while the effect was disadvantageous when touch happened on the parental ramets in the shaded environment. In sum, the role of neighboring touch varied, relying on the light environment, which complicated the plant–plant interactions under dense vegetation. The nuanced interactions between neighboring touch and light conditions highlight the complexity inherent in the dynamics of plant development within dense vegetation. Our research contributes to understanding the growth dynamics of understory plants. Given that this study was conducted in a controlled environment, there may be discrepancies between the experimental conditions and natural conditions. Therefore, future research should explore conducting field experiments to bridge this gap.

## Data availability statement

The original contributions presented in the study are included in the article/**Supplementary Material**. Further inquiries can be directed to the corresponding author.

## Author contributions

LX: Formal analysis, Writing – review & editing, Data curation, Investigation, Writing – original draft. JQ: Data curation, Formal analysis, Writing – review & editing. SZ: Writing – review & editing. XL: Writing – review & editing, Formal analysis, Funding acquisition, Methodology, Project administration, Resources, Supervision. HB: Writing – review & editing. MY: Writing – review & editing.
